# A thirteen-million-year divergence between two lineages of Indonesian coelacanths

**DOI:** 10.1038/s41598-019-57042-1

**Published:** 2020-01-13

**Authors:** Kadarusman, Hagi Yulia Sugeha, Laurent Pouyaud, Régis Hocdé, Intanurfemi B. Hismayasari, Endang Gunaisah, Santoso B. Widiarto, Gulam Arafat, Ferliana Widyasari, David Mouillot, Emmanuel Paradis

**Affiliations:** 1 Politeknik Kelautan dan Perikanan Sorong, KKD BP. SR SGK, Jl. Kapitan Pattimura, Tanjung Kasuari, Kota Sorong, 98401 Papua Barat, Indonesia; 2 Sekolah Tinggi Perikanan, KKD Pengelolaan Sumberdaya Perairan. SR BE, Jl. AUP, Pasar Minggu, Jakarta, 12520 Indonesia; 3 Research Center for Oceanography, Indonesian Institute of Sciences, Jl. Pasir Putih 1, Ancol Timur, Jakarta, 14430 Indonesia; 4 ISEM, Univ Montpellier, CNRS, EPHE, IRD, Montpellier, France; 5 MARBEC, Univ Montpellier, IRD, Ifremer, CNRS, Montpellier, France; 6 Loka Pengelolaan Sumberdaya Pesisir dan Laut Sorong, Jl. KPR PDAM, Km. 10, Kota Sorong, 98416 Papua Barat, Indonesia

**Keywords:** Molecular evolution, Phylogenetics

## Abstract

Coelacanth fishes of the genus *Latimeria* are the only surviving representatives of a basal lineage of vertebrates that originated more than 400 million years ago. Yet, much remains to be unveiled about the diversity and evolutionary history of these ‘living fossils’ using new molecular data, including the possibility of ‘cryptic’ species or unknown lineages. Here, we report the discovery of a new specimen in eastern Indonesia allegedly belonging to the species *L*. *menadoensis*. Although this specimen was found about 750 km from the known geographical distribution of the species, we found that the molecular divergence between this specimen and others of *L*. *menadoensis* was great: 1.8% compared to 0.04% among individuals of *L*. *chalumnae*, the other living species of coelacanth. Molecular dating analyses suggested a divergence date of *ca*. 13 million years ago between the two populations of Indonesian coelacanths. We elaborate a biogeographical scenario to explain the observed genetic divergence of Indonesian coelacanth populations based on oceanic currents and the tectonic history of the region over Miocene to recent. We hypothesize that several populations of coelacanths are likely to live further east of the present capture location, with potentially a new species that remains to be described. Based on this, we call for an international effort to take appropriate measures to protect these fascinating but vulnerable vertebrates which represent among the longest branches on the Tree of Life.

## Introduction

**Figure 1:**
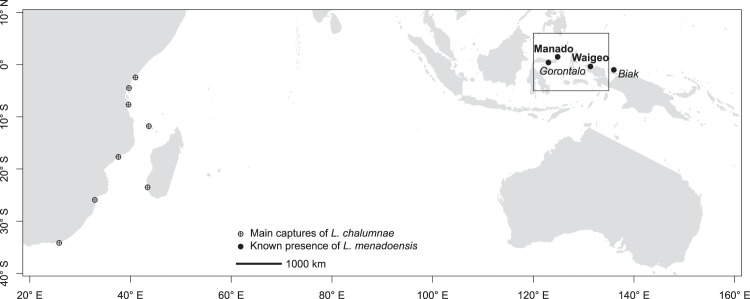
Known geographical distributions of the two species of *Latimeria*. The localities where specimens of *L*. *menadoensis* were captured are shown in bold, and the localities where this species has been observed *in situ* are in italics. The rectangle shows the limits of Fig. 5.

Coelacanth fishes of the genus *Latimeria* are the only living representatives of the Actinistia, a group of vertebrates that flourished between the Early Devonian (*ca*. 400 millions of years ago, Ma) and the Late Cretaceous (*ca*. 80 Ma)^1–3^. Fossil fishes belonging to the subclass Actinistia have been found around the world, whereas the two known living species of coelacanth are found only in the Western Indian Ocean (*L*. *chalumnae*^4^) and in Indonesia (*L*. *menadoensis*^5^). Sampling living specimens of coelacanths is a difficult endeavour, because they live at depths of 200 to 400 m although they occasionally occur at shallower depths of 100 m below the surface, while catches by fishermen are sporadic. Furthermore, direct observations suggest that coelacanths have a very sparse distribution and live in groups of a few tens of individuals^6–8^. Our current knowledge on the genetic diversity of these enigmatic animals suggests that *L*. *chalumnae* is distributed along several thousands kilometers of the South-East African coast and has very low genetic diversity based on mitochondrial genome markers^9,10^ and on nuclear microsatellites^11^, whereas *L*. *menadoensis* is distributed in a very small area near the northern part of the island of Sulawesi, where only a few individuals have been studied genetically but show very close genetic affinities^12,13^ (Fig. 1). These population genetic data strongly suggest that both species may represent the relicts of an ancient clade of early tetrapods decimated by extinctions^14^. Surprisingly, no specimens have been reported outside of these two restricted regions, while the existence of other coelacanth sub-species or species has never been mentioned.

We here report results of the genetic analyses of a new coelacanth specimen, allegedly belonging to *L*. *menadoensis*, caught recently in West Papua, Indonesia, approximately 750 km from Sulawesi. We find significant but Neogene-dated genetic differences between this individual and populations of *L*. *menadoensis* found in Sulawesi, challenging the view that living coelacanths are merely remnant populations of very anciently diverged lineages that have experienced little evolution since the early–mid Cenozoic.

## Results

**Figure 2:**
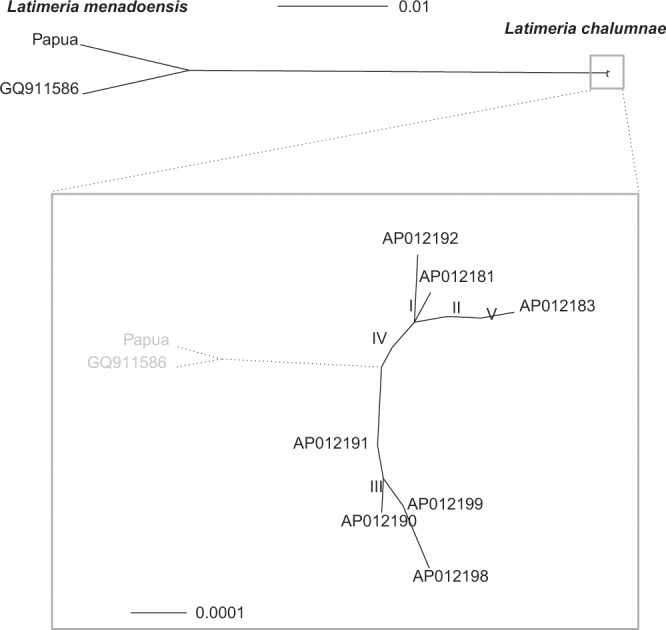
Maximum likelihood unrooted phylogeny reconstructed with 27 mitochondrial genome sequences of *Latimeria*. The Roman numerals indicate sequences that are identical (I: AP012182, AP012184–AP012187, AP012189, AP012193, and AP012195; II: AB257297, AP012179, AP012180, and AP012188; III: AB257296 and AP012177; IV: AP012178 and AP012196; and V: AP012194 and AP012197).

We sequenced 8164 base pairs (bp) of the mitochondrial genome of the new specimen, which represent 49.6% of the whole mitogenome of the *Latimeria* specimen from Manado (GQ911586, 16 446 bp). The alignement of these two sequences showed four deletions of one base (in tRNA-Phe, 16S rRNA, tRNA-Cys, and D-loop), and one deletion of two bases (D-loop) in the new specimen sequence, as well as two deletions of one base (in 16S rRNA and tRNA-Met) in GQ911586. This two-sequence alignment had thus 16 448 bp. The alignment with two genomes of *L*. *chalumnae* (AB257297, AP012199) added more gaps and resulted in an alignement with 16 454 bp. Alignments of 27 sequences of *Latimeria* spp., including sequences from previously sampled populations, generated with MUSCLE or with MAFFT (see Methods), confirmed this result. The inferred maximum-likelihood (ML) gene tree topology showed unambiguously that the two Indonesian coelacanths are sister-lineages with a level of divergence never observed among the individuals of *L*. *chalumnae* (Fig. 2). The bootstrap analysis gave the following 95% confidence intervals (CI) for the two terminal branch lengths leading to the Indonesian coelacanths: [0.008, 0.013] for the Papua sequence, and [0.007, 0.013] for GQ911586. The 95% CI for the length of the internal branch connecting the two Indonesian specimens to the *L*. *chalumnae* clade was [0.034, 0.042].

**Figure 3:**
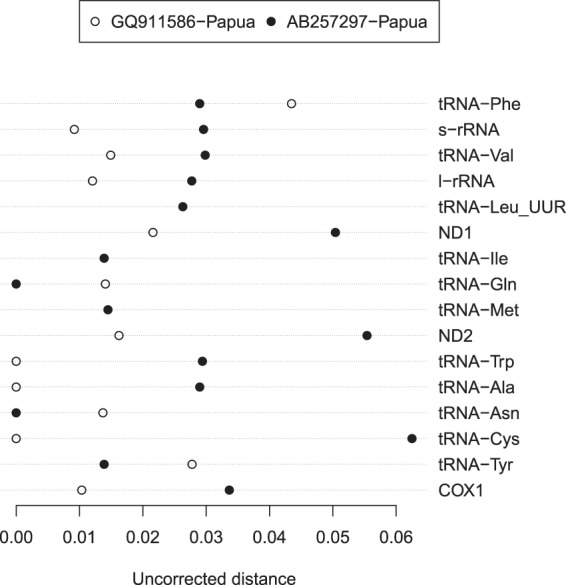
Uncorrected genetic distances for 16 genes of the mitogenome of *Latimeria*. Open symbols show distances between the two Indonesian specimens; filled symbols show distances between the new Indonesian specimen and a specimen of *L*. *chalumnae*.

The pairwise genetic distances among individuals of *L*. *chalumnae* were zero for all genes except for tRNA-GLn (0.014 between AB257297 and AP012199). In four cases (tRNA-Phe, tRNA-Gln, tRNA-Asn, and tRNA-Tyr), the distance between the two Indonesian specimens was greater than the distance between the new specimen and *L*. *chalumnae* (Fig. 3). In the twelve other cases, the opposite was observed, particularly for protein-coding and rRNA-coding sequences which are much longer than those coding for tRNAs. The p-distance between the two Indonesian specimens calculated using the first 655 sites of the COI gene (the segment used for fish DNA barcoding) was 0.0122.

In addition to the deletions noted above during alignment, the two Indonesian individuals showed 149 base differences, whereas the number of differences between two individuals of *L*. *chalumnae* varied between 0 and 10. Among the 13 proteins coded by the mitogenome, three had their genes sequenced in the new specimen: ND1, ND2, and COI. While no amino acid (AA) substitutions were observed among the 12 unique sequences of *L*. *chalumnae*, 35 AA replacements were observed between the Indonesian individuals and *L*. *chalumnae* (Table 1). Interestingly, it seems that all these replacements are the result of single events occurring on one of the three longest branches of the tree on Fig. 2. Among the 11 AA differences between the two Indonesian individuals, six can be inferred to result from substitutions that happened on the terminal branch leading to the new sequence, and five on the branch leading to GQ911586. The other 24 AA substitutions happened on the internal branch connecting the two main clades of *Latimeria*.

**Table 1:** Replacements of amino acids among the new coelacanth specimen from Papua (Papua), an individual from Manado (GQ911586), and an individual belonging to *L*. *chalumnae* (AP012199).

Sequence	Protein		
	ND1	ND2	COI
	000001112333	00001111122222222333	001
	177895786012	49990459902334489112	573
	489687616553	15688350194250368390	623
Papua	IAALYSTMVLLT	ITQTVFMVIITMTTMTYTTD	IIG
GQ911586	V...H.A....A	T..IMY...V..........	.VS
AP012199	VTTMHA.TIMF.	TAPI.YTMV.ATAATAHIPG	V..

The three rows of digits give the positions along the protein sequences and have to be read vertically.

**Figure 4:**
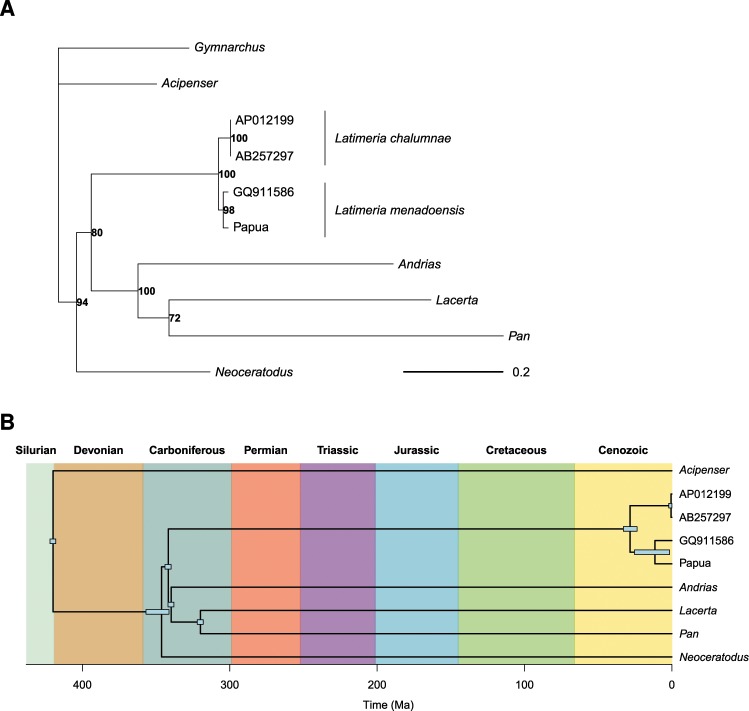
(**A**) Maximum likelihood phylogeny estimated with phangorn. The bold numbers give the node support bootstrap values (1000 replications). The branch lengths are in expected number of substitutions. (**B**) Dated phylogeny (chronogram) estimated by Bayesian inference. The rectangles show the credibility intervals from the highest posterior densities.

The ML phylogenetic analysis with ten mitogenomes resulted in an unambiguous tree that highlighted well-established relationships among the main clades of vertebrates (Fig. 4). A remarkable result from this phylogeny is the substantial variation in molecular evolutionary rate among sampled lineages. It is also noteworthy that *Pan* was found to be a sister-lineage of the clade *Andrias* + *Lacerta* in the NJ tree which was used as initial tree of the ML analysis.

The estimated divergence date for the two Indonesian coelacanth populations was inferred to be 13.3 Ma by the ML method, and 12.4 Ma by the Bayesian method (95% highest posterior density, HPD: 1.5–34.1), in the middle Miocene.

**Figure 5:**
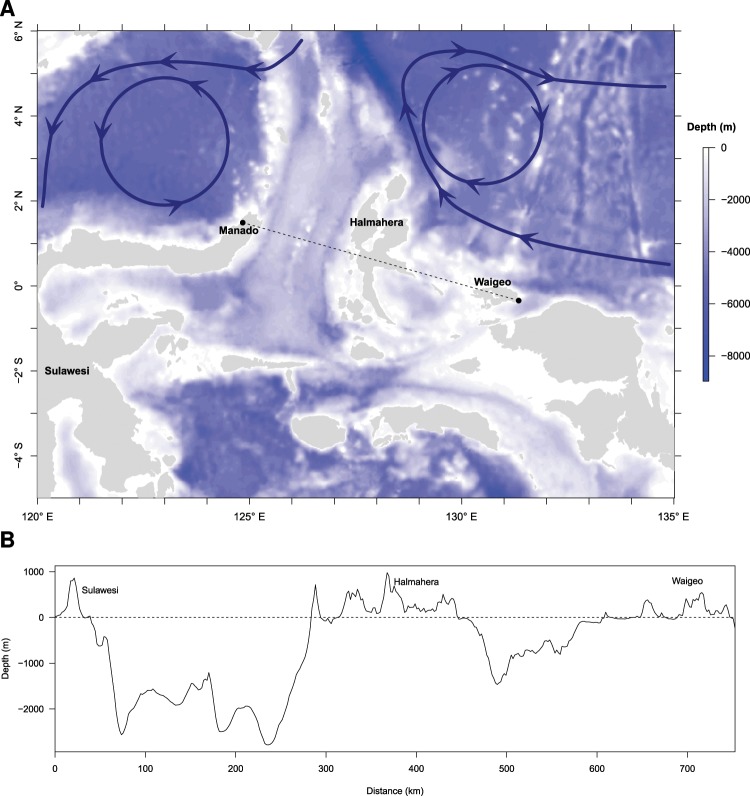
(**A**) Bathymetry of Eastern Indonesia. The arrows show the main surface currents. (**B**) Bathymetry and elevation profile between Manado and Waigeo.

**Figure 6:**
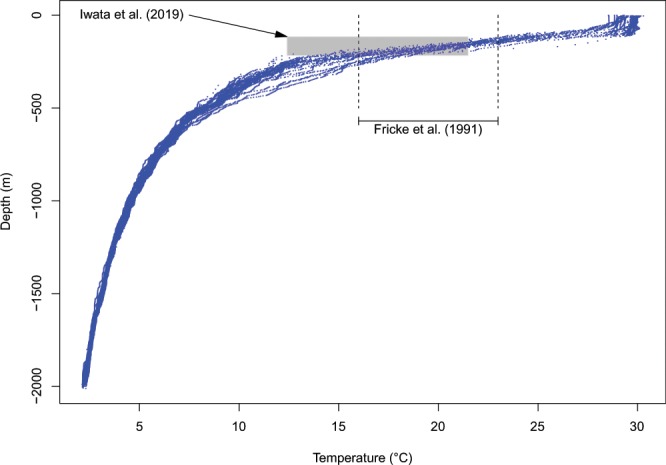
Temperatures and depths recorded close to the capture location of the new specimen (blue dots). The dashed lines show the depth range observed for *L*. *chalumnae* by Fricke *et al*.^6^, and the grey rectangle shows the depth and temperature ranges observed by Iwata *et al*.^8^ for *L*. *menadoensis*.

The distance between Manado (where all individuals of *L*. *menadoensis* were caught until the new specimen was captured, although some individuals were observed elsewhere; see Discussion section) and the location of the capture of the new individual was estimated to be 752 km (geodesic distance). The bathymetry around these locations is extremely variable with trenches more than 8000 m deep as well as shallow oceanic shelves (Fig. 5). Manado and Waigeo are separated by the island of Halmahera and a deep channel separates this island from Sulawesi. We extracted 25 724 temperature records taken at depth between 0 and 2011 m north of Papua. *Latimeria chalumnae* is reported to prefer water temperatures ranging from 16 °C to 23 °C^6^, and these temperatures were observed in our data at depths between 110 m and 270 m (Fig. 6).

## Discussion

Before the capture of the new *Latimeria* specimen reported in this paper, all known specimens of *L*. *menadoensis* were captured near Manado in North Sulawesi^8^. However, submarine observations of coelacanths have been reported 360 km southwest of Manado^15^ and in the Cendrawasih Bay to the north of Papua^8^, indicating potential for undetected populations. Therefore, our findings provide an important contribution to better understand the diversity, distribution, and evolution of this important group of vertebrates^16–18^.

We acknowledge several limitations of our study with regard to relying on a mtDNA-only dataset from a single individual of the new population. For example, examining loci from both nuclear and mitochondrial genomes has revealed cases of cytonuclear discordance in other taxonomic groups^19–21^. In the current investigation, we may have detected a divergent mitochondrial lineage in *L*. *menadoensis*. However, nuclear loci may reveal contemporary gene flow between populations from Manado and Papua New Guinea. Sampling additional specimens and expanding future molecular investigations to include unlinked nuclear loci would extend our understanding of species limits and the evolutionary history of *Latimeria*. However, the most striking result from our analyses is the relatively deep but Neogene molecular divergence between the specimen from Papua and its counterpart representing the previously studied population from Manado. This divergence is apparent at the mitochondrial DNA level but also implied a substantial number of AA replacements in protein-coding mitochondrial genes, something that has not to-date been observed among *L*. *chalumnae* individuals. The ML bootstrap analysis of branches leading to the Indonesian coelacanth fish populations strongly suggests that molecular evolutionary rate was equal in these two lineages, thus supporting the hypothesis of a molecular clock for the mitochondrial genome within this clade. Furthermore, the number of amino acid substitutions were found to be almost the same on both branches (five and six, respectively).

This marked pattern of genetic divergence between the two Indonesian coelacanths suggests that it would be worthwhile to revisit an earlier hypothesis^15^, namely that the two known species of *Latimeria* have different habitats and probably different life-history strategies that could be correlated to differences in their respective environments. East African coelacanth fish live in relatively calm oceanic waters while Indonesian coelacanth fish inhabit more disturbed and turbulent waters^7,15^. The eastern part of the Indonesian archipelago is characterized by a complex system of strong oceanic currents^22,23^. This complexity is partly caused by the meeting of several currents at the equator, where the Coriolis force becomes inverted. Particularly, the New Guinea Coastal Current (shown on the right-hand side of Fig. 5A) flows westward along the main coast of New Guinea, then northward after the island of Halmahera and finally eastward a few hundred kilometers further north. This phenomenon creates a whirlpool known as the Halmahera Eddy in the center of this loop with a depression of up to 120 m^24^. A similar phenomenon exists around Manado with the Mindanao Current (on the left-hand side of Fig. 5A) and the Mindanao Eddy west of Manado. These sea surface currents flow between 0 and 350 m below sea surface. The Maluku Sea appears to separate both populations of Indonesian coelacanths. These oceanographic observations agree with the existence of a strong barrier between Sulawesi and Halmahera which has been observed in several groups of marine organisms^25,26^.

A crucial question, given these results, is whether this system shaped by strong sea surface currents has been stable over geological time, to maintain the divergence of coelacanth populations over thousands to millions of years. Modelling studies suggest that the contrasted oceanic topography is an important factor in this region where waters from the northern and southern hemispheres meet^23,24^. This topography is the result of a long and complex tectonic history^27,28^. One of the most dramatic geological events was the uplift of New Guinea by the accretion of terranes (pieces of tectonic plates) with different plates and arcs between 42 Ma and 18 Ma^29^. The climax of changes in plate boundaries seem to have happened between about 25 and 20 Ma^27^. Therefore, this hydrological connectivity among marine populations or species was still possible before 20 Ma and was probably interrupted after this date. Our molecular dating analysis is consistent with these geological inferences and supports the interpretation of major barriers to connectivity between New Guinea and Sulawesi existing since around 20 Ma in the early- to mid-Miocene.

The molecular divergence observed between the individuals from Waigeo and Manado is further evidence of a low rate of molecular evolution among the lineages of *Latimeria*. Remarkably, such a low rate of molecular evolution was observed in previous studies comparing the two species using mitochondrial^13^ or nuclear genomes^30^. The observed divergence for the COI-based barcode between the two Indonesian specimens (1.22%) was greater than among individuals of the same species for several groups of fishes which averaged 0.59%^31^, 0.89%^32^, or 0.29%^33^. This suggests that the new specimen is genetically distinct and may warrant formal recognition as a species, although in-depth taxonomic assessment would be required to test this hypothesis.

Iwata *et al*.^8^ performed 1173 dives in Sulawesi and Biak and observed 30 coelacanths at depths 115.6–218.9 m and temperatures 12.4–21.5 °C (Fig. 6). These values widely overlap with those observed for *L*. *chalumnae*^6^, though depths for the latter are slightly shallower. It is too premature to conclude that the temperature tolerances of these species are different, although the relatively old divergence between them (30–40 Ma) is likely to result in environmental niche divergence through time.

Based on the available information from our results, in combination with patterns of oceanic circulation from the region, we hypothesize that the *Latimeria* specimen captured in Waigeo in July 2018 belongs to *L*. *menadoensis*, although it is likely that this specimen belongs to a distinct and as-of-yet undescribed species or subspecies of *Latimeria*. We urgently call for future taxonomic studies integrating morphological and genetic data from multiple specimens from different *L*. *menadoensis* populations, in order to test this hypothesis. Nevertheless, our results suggest that *L*. *menadoensis* likely has a much wider geographical range than previously thought. Given the presence of this specimen nearby the New Guinea Coastal Current, we hypothesize that *L*. *menadoensis* populations can occur along the coast of New Guinea and probably further east of New Guinea. The waters of the northwest coast of New Guinea have been poorly sampled and it would not be surprising to find several populations of coelacanths in the region.

In light of our findings as well as the unique biology of coelacanths in general, we call for an international effort to take appropriate measures to protect these fascinating but vulnerable vertebrates. The conservation of relict species including the unique representatives of particular phylogenetic groups and their likely unique combinations of characters is key to conserve phylogenetic diversity represented by the Tree of Life. Yet, the existing global system of marine protected areas (MPAs) may fail to cover areas where these relict species are located^34^. If MPAs are to protect the unique and diverse marine lineages across the Tree of Life, we need to carefully reconsider the protection of East Indonesian coasts where this new lineage of coelacanth occurs.

## Methods

### Sampling

Mr. Dava Santoso, a recreational fisherman, accidentally caught a coelacanth on July 1, 2018, off southeast Waigeo Island (West Papua, Indonesia; Fig. 5A). The specimen was captured at a depth, estimated by Mr. Santoso, of about 300 m with a handline while using sardine fillets as bait. The gender of the specimen was not identified, and the body was around 1 m long. Unfortunately, the specimen was filleted and mostly consumed before the staff from Loka Pengelolaan Sumberdaya Pesisir dan Laut Sorong (LPSPL Sorong, Indonesia) and the Politeknik Kelautan dan Perikanan Sorong (Politeknik KP Sorong) obtained tissue samples from the fish and preserved them in absolute ethanol (100%).

### Laboratory analyses

Laboratory analyses were carried out at the molecular laboratory of the Ministry of Marine and Fisheries Affairs in Jakarta, Indonesia. Whole genomic DNA was extracted using ZR Tissue & Insect DNA MiniPrep (Zymo Research, D6016). We amplified half of the whole mitochondrial genome including the whole control region (D-loop), 12S, 16S, ND1, ND2, and COX1, using 12 pairs of primers described in^13^ (Table S1) with an initial denaturation step at 94 °C for 2 min, a cycle of three steps (denaturation at 94 °C for 30 sec; annealing at 55 °C for 40 sec; elongation at 72 °C for 1 min) repeated 35 times, and a final extension of 10 min at 72 °C. The double-stranded PCR products were purified and sequenced using the BigDyeTM Terminator v3.1 Cycle Sequencing Kit at 1st BASE Manufacturer, Axil Scientific Pte Ltd., Singapore.

### Statistical analyses

We first compared the new mitogenome sequence with the sequence from an individual of *L*. *menadoensis* caught in Manado (GenBank accession no: GQ911586^13^) and 25 sequences of *L*. *chalumnae* from Comoros (2) and Tanzania (23) (AB257296, AB257297, AP012177–AP012198^9,10^). We performed manual sequence alignments as well as alignments with MUSCLE^35^ and MAFFT^36^. We examined the base substitutions as well as the amino acid substitutions for the protein-coding sequences with ape^37^, and performed maximum likelihood (ML) phylogenetic analyses under a GTR + Γ + I model using the phangorn R package^38^. Confidence in the inferred clades was assessed using a bootstrap approach with 1000 pseudoreplicates^39^. Because we used a general phylogenetic model with a distinct substitution rate for each branch (i.e., not a molecular clock model), we tested the hypothesis that the rate of molecular evolution was the same between specimens of Indonesian *Latimeria* using a bootstrap with 1000 pseudoreplicates and comparing the distribution of these estimates. This test makes no assumption on the substition rates on the other branches of the tree. The uncorrected genetic distances (also known as p-distances) were calculated separately for 16 sequences coding for tRNAs (11), rRNAs (2), or proteins (3) using annonations from GenBank.

Subsequent to the above steps, we dated the divergence between the two specimens of *Latimeria* found in Indonesia by reconstructing a phylogeny with the mitogenomes of several vertebrates, including the followings: *Acipenser dabryanus* (KP981414), *Gymnarchus niloticus* (AP008930), *Neoceratodus forsteri* (AJ584642), *Andrias japonicus* (AB208679), *Lacerta bilineata* (NC_028440), and *Pan troglodytes* (NC_001643). These species were selected in order to provide a reasonable sample whose genomes and relationships to Actinistia are well characterized^40,41^. A progressive alignment of these six additional genomes was performed on the alignement obtained with the new sequence, GQ911586, AB257297, and AP012199 using MAFFT. An ML tree was estimated with this alignment under a GTR + Γ + I model and then dated with the ML method^42^ implemented in ape as well as an independent analysis using the Bayesian approach implemented in BEAST 2.5.2^43^. The ML analysis used Sanderson’s correlated rate model where the substitution rates are assumed to be positively correlated on contiguous branches of the tree^44^, whereas the Bayesian analysis assumed a relaxed lognormal clock model and Yule priors on the distribution of trees. Three calibration points were used in both analyses^41^: 416.1–421.75 Ma for the divergence between fishes and tetrapods (Actinopterygii–Sarcopterygii), 330.4–350.1 Ma for the divergence between amniotes and amphibians (Reptiliomorpha–Batrachomorpha), and 312.3–330.4 Ma for the divergence between reptiles and mammals (Sauropsida–Synapsida). These ages were considered as soft bounds in both analyses using normal priors in Bayesian analysis. The posterior distribution of the parameters were estimated by Markov chain Monte Carlo (MCMC) run during 10^7^ generations sampled every 10^3^ and discarding the first 10^6^ generations as burn-in period.

In order to assess potential dispersal routes between Manado and Waigeo, we established the bathymetric profile of the region using the GIS database provided by the National Oceanic and Atmospheric Administration (https://www.ngdc.noaa.gov/mgg/global/relief/ETOPO1/data/bedrock/grid_registered/georeferenced_tiff/, accessed 2019-03-18). These data were processed with the R package rgdal^45^. Because no vertical ocean profiles of temperature data are available on the location of the capture of the new specimen, we used data from the closest stations which are made available by the ARGO broad-scale global array of temperature and salinity profiling floats in the northeast of Waigeo. The area is crossed by the Halmahera Eddy throughflow from the Pacific Ocean to the Indian Ocean. We can therefore consider that the characteristics of the seawater column observed by the selected ARGO profiling floats are similar to those in Waigeo. ARGO is an international program that calls for the deployment of 3,000 free drifting profiling floats, distributed over the global oceans which measure the temperature and salinity in the upper 2 000 m of the ocean. The observation data are uploaded in the Global Data Assembly Centre (Argo GDAC^46^). Each ARGO autonomous profiling CTD has several sensors on board, including a Sea-Bird SBE 41/41CP for the seawater temperature and salinity acquisition, and a Druck 2900 PSIA for the pressure acquisition. Argo CTD module was developed in response to the scientific need for highly stable and accurate temperature and salinity on profiling floats, with high and manual quality control process^47^. The seawater temperature profiles were extracted from the ARGO database (http://www.ifremer.fr/co-argoFloats/float?ptfCode=5904515) with an accuracy of 0.002 °C and a resolution of 0.0001 °C for the temperatures, and an accuracy of 1.5% and a resolution 0.01 dbar for the pressure. We extracted 209 profiles recorded during March 2019 at longitudes between 141.02°E and 161.21°E and latitudes between 8.18 °S and 1.67 °N.

Except for the BEAST analysis, all analyses were done with R version 3.5.2^48^.

## Supplementary information

Supporting Information.

## Data Availability

The new sequence was deposited in NCBI GenBank (accession number: MK748470).
