# Publisher Correction: A thirteen-million-year divergence between two lineages of Indonesian coelacanths

**DOI:** 10.1038/s41598-020-61557-3

**Published:** 2020-03-10

**Authors:** Kadarusman, Hagi Yulia Sugeha, Laurent Pouyaud, Régis Hocdé, Intanurfemi B. Hismayasari, Endang Gunaisah, Santoso B. Widiarto, Gulam Arafat, Ferliana Widyasari, David Mouillot, Emmanuel Paradis

**Affiliations:** 1 Politeknik Kelautan dan Perikanan Sorong, KKD BP. SR SGK, Jl. Kapitan Pattimura, Tanjung Kasuari, Kota Sorong, 98401 Papua Barat, Indonesia; 2 Sekolah Tinggi Perikanan, KKD Pengelolaan Sumberdaya Perairan. SR BE, Jl. AUP, Pasar Minggu, Jakarta, 12520 Indonesia; 3 Research Center for Oceanography, Indonesian Institute of Sciences, Jl. Pasir Putih 1, Ancol Timur, Jakarta, 14430 Indonesia; 4 ISEM, Univ Montpellier, CNRS, EPHE, IRD, Montpellier, France; 5 MARBEC, Univ Montpellier, CNRS, Ifremer, IRD, Montpellier, France; 6 Loka Pengelolaan Sumberdaya Pesisir dan Laut Sorong, Jl. KPR PDAM, Km. 10, Kota Sorong, 98416 Papua Barat, Indonesia

Correction to: *Scientific Reports* 10.1038/s41598-019-57042-1, published online 13 January 2020

In the original version of this Article, Figure 1 contained an error where Northeastern Australia was omitted.

In addition, there was a typographical error in Affiliation 5, which was incorrectly given as ‘MARBEC, Univ Montpellier, IRD, Ifremer, CNRS, Montpellier, France’. The correct affiliation is listed below:

MARBEC, Univ Montpellier, CNRS, Ifremer, IRD, Montpellier, France

These errors have now been corrected in the HTML and PDF versions of the Article.

