# Supplementary material for: A thirteen-million-year divergence between two lineages of Indonesian coelacanths
Source: Sci Rep. 2020 Jan 13;10:192. doi: 10.1038/s41598-019-57042-1 (PMC6957673; doi:10.1038/s41598-019-57042-1)
Supplement: Supplementary file 1 — Supporting Information. [file 41598_2019_57042_MOESM1_ESM.pdf]

A thirteen-million-year divergence between two lineages of Indonesian coelacanths

Kadarusman, Hagi Yulia Sugeha, Laurent Pouyaud, Régis Hocdé, Intanurfemi B. Hismayasari, Endang Gunaisah, Santoso B. Widiarto, Gulam Arafat, Ferliana Widyasari, David Mouillot, Emmanuel Paradis

Supporting Information

Table S1: Pairs of primers used for the PCRs (the sequences are in [1]).

Gene/region amplified	Primer pairs	
D-loop	CYTBF3	DLOOPR1
	DLOOPF1	12SR1
12S	12SF1	12SR2
	12SF2	16SR1
16S	16SF1	16SR2
	16SF2	ND1R1
ND1	ND1F1	TRILER1
	ND1F2	ND2R1
ND2	ND2F1	ALAR1
COX1	ALAF1	COX1R1
	COX1F1	COX1R2
	COX1F2	COX2R1

[1] Sudarto *et al.* Mitochondrial genomic divergence in coelacanths (*Latimeria*): slow rate of evolution or recent speciation? Marine Biology **156**, 2253–2262 (2010).

R Script

```
## script associated with the manuscript: "A thirteen-million-year
## divergence between two lineages of Indonesian coelacanths"
## by Kadarusman et al. (submitted)

library(ape)

## NOTE: the present script requires MUSCLE and MAFFT to be installed, and
## is expected to run 'as is' on a Linux (Ubuntu) system.

## first manual alignemnt with four sequences:
x <- read.dna("coelacanthcomplet.fas", "f")
rownames(x) <- c("Papua", "GQ911586", "AB257297", "AP012199")

## add other sequences from GenBank:
ext <- read.GenBank(c("AB257296", paste0("AP0121", 77:98)))

## do progressive alignement with MUSCLE:
xx <- muscle(x, ext, quiet = FALSE)

## do alignement from scratch with MAFFT:
library(ips)
xx2 <- mafft(c(del.gaps(x), ext), path = "/usr/bin/mafft", quiet = FALSE)
## compare both alignments:
all.equal.DNAbin(xx, xx2, plot = TRUE)

## get the haplotypes
library(pegas)
h <- sort(haplotype(xx))
plot(h)
index <- attr(h, "index")
n <- length(index)

## make new labels:
newlabs <- character(n)
for (i in 1:n) {
    idx <- index[[i]]
    if (length(idx) == 1) newlabs[i] <- labels(xx)[idx]
    else {
        newlabs[i] <- as.character(as.roman(i))
    }
}
## build the matrix to be used for the ML analysis:
X <- h[]
attr(X, "index") <- attr(X, "from") <- NULL
```

```
rownames(X) <- newlabs

## make NJ tree:
d <- dist.dna(X)
trnj <- nj(d)

## do the ML analysis:
library(phangorn)
z <- as.phyDat(X)
m0 <- pml(trnj, z)
m1 <- optim.pml(m0, optNni = TRUE, optQ = TRUE, optBf = TRUE)
m1g <- optim.pml(update(m1, k = 4), optGamma = TRUE, optInv = TRUE,
                 optQ = TRUE, optBf = TRUE, optNni = TRUE)
anova(m1, m1g) # P = 0.003

## extract the ML tree:
trml <- m1g$tree

## do bootstrap to get confidences intervals on the 3 branch lengths
## leading to the Indonesian specimens

TR.ML <- bootstrap.pml(m1g, 1000)

## we can safely assume that in all 1000 bootstrap trees the longest
## branch is the one connecting the two species of Latimeria:
quantile(sapply(TR.ML, function(x) max(x$edge.length)), c(0.025, 0.975))
##       2.5%      97.5%
## 0.03381317 0.04230008

## for the two terminal branches leading to the two specimens from Indonesia,
## we build a small function to get their lengths (could be written on a
## single line but would be less clear):
f <- function(x, lab) {
    i <- which(x$tip.label == lab)
    b <- which(x$edge[, 2] == i)
    x$edge.length[b]
}
quantile(sapply(TR.ML, f, lab = "Papua"), c(0.025, 0.975))
##        2.5%       97.5%
## 0.007539421 0.012958961
quantile(sapply(TR.ML, f, lab = "GQ911586"), c(0.025, 0.975))
##       2.5%      97.5%
## 0.00740426 0.01254890

################################################################
## amino acid replacements
```

```
## from GenBank GQ911586 (see Sudarto et al. 2010):
tab <- read.table("~/data/Projets/Latimeria/Sweave/mtGenome.txt",
                  header = TRUE, row.names = 3)

## reorder the sequences:
X <- X[6:8, ]

for (prot in c("ND1", "ND2", "COX1")) {
    cat("***", prot, "***\n")
    offset <- switch(prot, "ND1" = 4, "ND2" = 5, "COX1" = 10)
    a <- tab[prot, "start"]
    b <- tab[prot, "end"]
    if (prot == "COX1") b  <- b - 3
    print(dnds(X[, a:b + offset], code = 2, quiet = TRUE))
    y <- trans(X[, a:b + offset], 2)
    ## print(dist.aa(y))
    j <- AAsubst(y)
    cat("\n")
    cat(paste0("          ", .showpos(j)), sep = "\n")
    alview(y[, j])
    ## dna <- X[, a:b + offset]
    ## m <- matrix(1:(ncol(y)*3), ncol = 3, byrow = TRUE)
    ## alview(dna[, as.vector(t(m[j, ]))])
}

.showpos <- function(x) {
    x <- as.integer(x)
    n <- length(x)
    digits <- floor(log10(x[n])) + 1
    res <- sprintf(paste0("%0", digits, "d"), x)
    res <- unlist(strsplit(res, ""))
    dim(res) <- c(digits, n)
    apply(res, 1, paste, collapse = "")
} # included in ape 5.3

#################################################################
## ANALYSIS WITH 10 SPECIES

ext2 <- read.GenBank(c("KP981414", "AP008930", "AJ584642", "AB208679",
                       "NC_028440", "NC_001643"))
names(ext2) <- c("Acipenser", "Gymnarchus", "Neoceratodus", "Andrias",
                 "Lacerta", "Pan")
## xx2 <- clustal(ext2, x, quiet = FALSE) # NOT GOOD
library(ips)
xx2 <- mafft(c(ext2, del.gaps(x)), path = "/usr/bin/mafft", quiet = FALSE)
```

```
checkAlignment(xx2)
saveRDS(xx2, "xx2.rds")

trnj2 <- nj(dist.dna(xx2))
og <- c("Acipenser", "Gymnarchus")

layout(matrix(1:2, 1))
plot(dist.dna(xx2, "raw"), dist.dna(xx2)); abline(0, 1, lty = 3)

plot(trnj2, "u", lab4ut = "a", label.offset = 0.005)
plot(root(trnj2, og), lab4ut = "a", label.offset = 0.005)

library(phangorn)
z <- as.phyDat(xx2)
m0 <- pml(trnj2, z)
m1 <- optim.pml(m0, optNni = TRUE, optQ = TRUE, optBf = TRUE)
m1g <- optim.pml(update(m1, k = 4), optGamma = TRUE, optInv = TRUE,
                 optQ = TRUE, optBf = TRUE, optNni = TRUE)
anova(m1, m1g) # P < 0.0001

trml2 <- root(m1g$tree, og)
saveRDS(trml2, "trml2.rds")

## from Benton & Donoghue 2007 (dates are in the paper):
cal <- read.delim("cal_Benton2007.dat")
MAX <- max(cal[c("age.min", "age.max")])
cal[, 2:3] <- cal[, 2:3]/MAX

trml2 <- readRDS("trml2.rds")
rtr <- drop.tip(trml2, og[2])
cal <- makeChronosCalib(rtr)

chr <- chronos(rtr, calibration = cal)
chr$edge.length <- chr$edge.length * MAX
rates <- attr(chr, "rates") / MAX
plot(chr, edge.width = 2000 * rates, label.offset = 5)

################################################################
## BEAST2 analysis

xx2 <- readRDS("xx2.rds")
setwd("beast/bin/")

system("./beauti &") # better to launch in a console

system("./beast -overwrite PAPUA.xml")
```

```
TR <- read.nexus("xx2b.trees")
N <- length(TR)
plot(TR[[N]]); axisPhylo()
branching.times(TR[[N]]); nodelabels()

apply(sapply(TR, branching.times), 1, summary)
apply(sapply(TR, branching.times), 1, quantile, probs = c(0.025, 0.975))
hist(sapply(TR, branching.times)["15", ])

#################################################################
## ARGO data
## http://www.ifremer.fr/co-argoFloats/float?ptfCode=5904515

od <- setwd("DataSelection_20190401_122616_7856498/")
fls <- dir()
profiles <- grep("profiles", fls, value = TRUE)

VARS <- c("PRES_ADJUSTED..decibar.", "TEMP..degree_Celsius.")#,
#           "DATE..yyyy.mm.ddThh.mi.ssZ.")#, "LATITUDE..degree_north.",
#           "LONGITUDE..degree_east.")
X <- NULL
for (fl in fls) {
    if (fl == "readme.txt") next
    tmp <- read.csv(fl, row.names = NULL)
    if (all(VARS %in% names(tmp))) X <- rbind(X, tmp[VARS])
}
dim(X) # [1] 32278     2
sapply(X, range, na.rm = TRUE)
##      PRES_ADJUSTED..decibar. TEMP..degree_Celsius.
## [1,]                    -1.5                 2.125
## [2,]                  2011.1                35.862

plot(X)

## subset the data
d <- is.na(X[, 1]) | is.na(X[, 2]) | X[, 2] > 31
d <- c(224, which(d))
Xb <- X[-d, ]
dim(Xb) # [1] 25724     2
plot(X, pch = ".", col = "blue")
ch <- chull(Xb)
polygon(Xb[ch, ])

abline(h = c(16, 23), lty = 3)
locator(2) # 110--270 m
```

```
## from Iwata et al. 2019:
rect(115.6, 12.4, 218.9, 21.5, border = rgb(.5, .5, .5, .15),
     col = rgb(.5, .5, .5, .15))
abline(v = c(115.6, 218.9), lty = 2)
abline(h = c(12.4, 21.5), lty = 2)

legend("topright", legend = c("Fricke et al. (1991)",
       "Iwata et al. (2019)"), lty = c(3, 2))
dev.copy2pdf(file = "rangeTD.pdf") # Fig. S2
```
